# Fusion of Mature HIV-1 Particles Leads to Complete Release of a Gag-GFP-Based Content Marker and Raises the Intraviral pH

**DOI:** 10.1371/journal.pone.0071002

**Published:** 2013-08-09

**Authors:** Sergi Padilla-Parra, Mariana Marin, Nivriti Gahlaut, Rolf Suter, Naoyuki Kondo, Gregory B. Melikyan

**Affiliations:** 1 Division of Pediatric Infectious Diseases, Emory University Children’s Center, Atlanta, Georgia, United States of America; 2 Children’s Healthcare of Atlanta, Atlanta, Georgia, United States of America; University of Missouri, United States of America

## Abstract

By imaging the release of a GFP-based viral content marker produced upon virus maturation, we have previously found that HIV-1 fuses with endosomes. In contrast, fusion at the cell surface did not progress beyond a lipid mixing stage (hemifusion). However, recent evidence suggesting that free GFP can be trapped within the mature HIV-1 capsid raises concerns that this content marker may not be released immediately after the formation of a fusion pore. To determine whether a significant portion of GFP is trapped in the mature capsid, we first permeabilized the viral membrane with saponin. The overwhelming majority of pseudoviruses fully released GFP while the remaining particles exhibited partial loss or no loss of content. The extent of GFP release correlated with HIV-1 maturation, implying that incomplete Gag processing, but not GFP entrapment by mature capsids, causes partial content release. Next, we designed a complementary assay for visualizing pore formation by monitoring the intraviral pH with an additional pH-sensitive fluorescent marker. The loss of GFP through saponin-mediated pores was associated with a concomitant increase in the intraviral pH due to equilibration with the pH of an external buffer. We next imaged single HIV-cell fusion and found that these events were manifested in a highly correlated loss of content and increase in the intraviral pH, as it equilibrated with the cytosolic pH. Fused or saponin-permeabilized pseudoviruses that partially lost GFP did not release the remaining content marker under conditions expected to promote the capsid dissociation. We were thus unable to detect significant entrapment of GFP by the mature HIV-1 capsid. Together, our results validate the use of the GFP-based content marker for imaging single virus fusion and inferring the sites of HIV-1 entry.

## Introduction

The HIV-1 Env glycoprotein initiates viral fusion with the host cell membrane through sequentially engaging CD4 and coreceptors, CXCR4 or CCR5 [1], [2]. The viral capsid is then released through a fully enlarged fusion pore into the cytoplasm where it undergoes uncoating and reverse transcription [3], [4]. HIV-1 fusion has long been thought to occur at the cell surface [5]–[10]. However, several lines of evidence imply that this virus enters target cells through endocytosis and fusion with endosomes [11]–[17]. Endocytic entry has also been implicated in HIV-1 cell-to-cell transmission [18]–[20], but this notion is not supported by other studies [21], [22].

We have previously investigated the sites of HIV-1 entry into cells using two complementary strategies [12]. First, kinetic measurements of virus-cell fusion revealed that HIV-1 acquires resistance to a membrane-impermeant peptide fusion inhibitor (targeting surface-accessible viruses) much earlier than to low temperature, which blocked all fusion events. This result suggests that HIV-1 escapes from inhibitory peptides, such as enfuvirtide [23], by entering an endocytic pathway and fusing with endosomes at a later time. Second, single virus fusion has been imaged using particles co-labeled with a GFP-based viral content marker and a lipophilic dye incorporated into the viral membrane. The fact that the viral content release was not associated with loss of a lipid marker demonstrated that full fusion occurred in small intracellular compartments that were not connected to the plasma membrane. In stark contrast, particles that exchanged lipids with the plasma membrane, as evidenced by quick disappearance of a viral membrane marker, rarely released their content. We found that HIV-1 exhibited a strong preference for endocytic entry, irrespective of the coreceptor tropism and of the choice of retroviral core used to produce pseudoviruses [12]. Our data also support endocytic HIV-1 entry into permissive adherent cells, T cell lines and primary CD4^+^ T cells [11], [12]. In addition, we have found that, upon blocking endocytosis, HIV-1 fusion [11], [24] and infection [17] were inhibited, while lipid mixing at the cell surface was exaggerated [11]. It thus appears that HIV-1 fusion with the plasma membrane is arrested at a hemifusion stage upstream of fusion pore formation.

Our single virus imaging experiments have primarily employed the Murine Leukemia Virus (MLV) Gag-GFP-labeled particles pseudotyped with the HIV-1 Env [11], [12]. The small nucleocapsid-GFP fragment produced upon Gag-GFP cleavage by the viral protease served as the viral content marker and was released through a fusion pore. In addition, we and others labeled particles with the HIV-1 Gag-iGFP construct originally made by Benjamin Chen’s lab [25]. Here, the “internal” GFP flanked by the protease cleavage sites is inserted between the MA (matrix) and CA (capsid) domains of Gag [12], [25]. The release of free GFP (hereafter referred to as iGFP) generated upon cleavage of Gag-iGFP enables the detection of viral fusion [12], [26].

**Figure 1:**
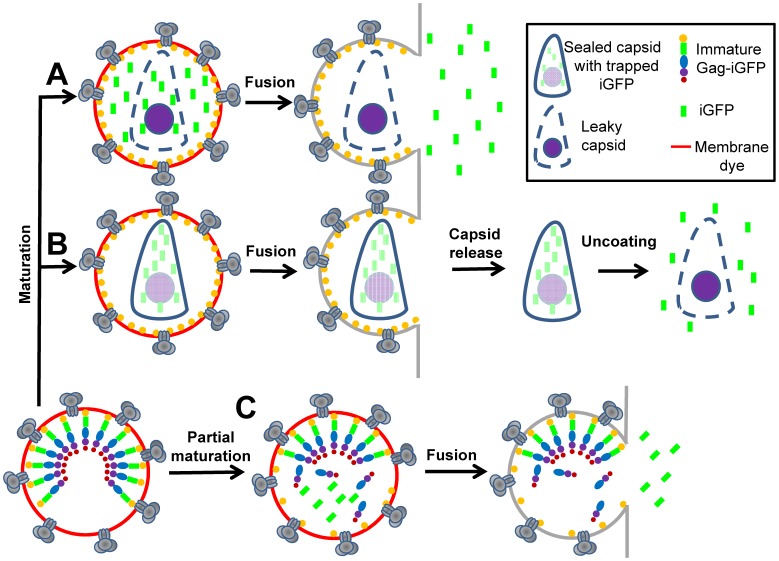
Models for fusion-mediated release of an HIV-1 content marker produced upon virus maturation. Shown are fully (A, B) and partially (C) matured particles originating from an immature HIV-1 virion (lower left). (A) A virus with mature “leaky” capsid fully releases the content marker (iGFP, green) through a fusion pore. (B) A sealed mature capsid may release iGFP after separating from the viral envelope and uncoating. An extreme case when all iGFP molecules are trapped within the capsid is illustrated. A more likely outcome of maturation is entrapment of a fraction of iGFP in the capsid. (C) A partially matured particle contains free iGFP that is released through a fusion pore, whereas the remnants of immature capsid (Gag-iGFP) are not released. Illustrated are the virus fusion events with the plasma membrane, whereupon the viral membrane marker (red) disappears as a result of dilution.

A recent study reported that iGFP released upon HIV-1 maturation could be trapped not only within the viral membrane, but also within the mature capsids [27]. Two lines of evidence supported this notion: (i) iGFP co-migrated with the purified capsids, and (ii) the majority of intact HIV-1 cores detected in the cytoplasm was GFP-positive. Intact post-fusion cores were identified based upon colocalization with the cytoplasmic bodies formed by TRIM5α, a restriction factor that recognizes the intact HIV-1 capsid [28], [29]. These results suggest that HIV-1 capsids can carry iGFP into the cytoplasm and that perhaps the loss of iGFP in live cell imaging experiments may report capsid uncoating, as opposed to the formation of a small fusion pore. In the extreme case that all iGFP molecules are trapped by the capsid (Fig. 1B), full fusion at the cell surface could be misinterpreted as hemifusion defined as lipid mixing without the viral content release. Although a fraction of iGFP within a mature virus should reside outside the capsid and thus be lost through a fusion pore (Fig. 1 and [12]), we nonetheless sought to test whether the full HIV-1 fusion with the plasma membrane can be missed due to the lack of an immediately releasable iGFP pool.

To validate Gag-iGFP as a marker for virus fusion, we modified the virus labeling strategy to enable independent detection of nascent pores in the viral membrane based on changes in the intraviral pH. Simultaneous measurements of pore formation by two independent approaches revealed that HIV-1 pseudoviruses released their content marker at the time of small pore opening. Most particles quickly lost the entire pool of iGFP, while others released a fraction of fluorescent protein or were fully resistant to lysis. The residual marker remaining after partial release did not escape from particles under conditions facilitating the capsid uncoating and was therefore likely caused by incomplete virus maturation (partial cleavage of Gag-iGFP). The immediate loss of the content marker through a nascent fusion pore and inverse correlation between HIV-1 maturation and retention of the GFP signal show that, under our conditions, the capsid-entrapped iGFP (if any) does not interfere with the detection of fusion pores. Our data thus demonstrate that loss of iGFP faithfully reports the formation of small pores in the viral membrane. We also conclude that partial content release or lack of release in a small fraction of pseudoviruses occur due to incomplete maturation and not to iGFP entrapment in the intact capsid.

## Materials and Methods

### Cell Lines, Reagents and Plasmids

Human embryonic kidney HEK293T/17 and African green monkey kidney CV-1 cells were obtained from ATCC (Manassas, VA). CV-1/TVA950 and TZM-bl/TVA950 cells expressing high levels of the TVA950 receptor for Avian Sarcoma and Leukosis Virus subtype A (ASLV-A) have been described previously [30]. CV-1 cells expressing CD4 and CXCR4 were kindly provided by Dr. D. Kabat [31]. HEK293T/17, TZM-bl, and CV-1/CD4/CXCR4 were grown in Dulbecco’s Modified Eagle high glucose medium (DMEM) (Cellgro, Manassas, VA) supplemented with 10% Fetal Bovine Serum (FBS, HyClone Laboratories, Logan, UT) and 100 U penicillin-streptomycin (Gemini Bio-Products, West Sacramento, CA). The growth medium for HEK293T/17 cells contained 0.5 µg/ml G418 (Cellgro). CV-1/TVA950 cells were grown in DMEM supplemented with 10% Cosmic Calf Serum (HyClone Laboratories, Logan, UT) and 100 U penicillin-streptomycin. DiD (1,1′-dioctadecyl-3,3,3′,3′-tetramethylindodicarbocyanine, 4-chlorobenzenesulfonate salt) was purchased from Invitrogen (Carlsbad, CA), Opti-MEM was from Gibco (Grand Island, NY), Hanks’ Balanced Salt Solution with calcium and magnesium (HBSS) was from Cellgro and poly-L-lysine was from Sigma (St. Louis, MO). The PF74 compound [32] was a gift from Dr. Chris Aiken (Vanderbilt University). The C52L recombinant peptide was a gift from Dr. Min Lu (New Jersey Medical School) [33]. The following reagents were obtained from the NIH AIDS Research and Reference Reagent Program (Division of AIDS, NIAID): indicator TZM-bl cells (donated by Drs. J.C. Kappes and X. Wu [34]), the HIV-1 protease inhibitor, saquinavir (SQV), the anti-p24 Hybridoma 183-H12-5C (donated by Dr. B. Chesebro [35]), and the pcRev vector expressing HIV-1 Rev [36]. The pCAGGS plasmid encoding the HXB2 envelope glycoprotein (Env) was provided by Dr. J. Binley (Torrey Pines Institute, CA) [37]. The HIV-1-based packaging vector pR8ΔEnv lacking the *env* gene was from Dr. D. Trono (EPFL, Lausanne, Switzerland). The YFP-Vpr and HIV-1 Gag-iGFP plasmids were gifts from Dr. T. Hope and Dr. B. Chen [25], respectively. Vectors expressing the ASLV-A Env glycoprotein lacking the cytoplasmic domain, and the ecliptic pHluorin-ICAM-1 chimera (designated EcpH-ICAM-1) have been described previously [38], [39]. Vectors expressing the MLV Gag-pol, MLV Gag-GFP, and the MLV LTR LacZ [40] were provided by Dr. W. Mothes (Yale University).

### Plasmids Construction

The HIV-1 Gag-iCherry plasmid was constructed by amplifying the mCherry gene by PCR using KOD Xtreme DNA polymerase (Novagen) and the following forward and reverse primers: *5′-* caaacggtaagggcgaggaggataacatggcc *-3′* and *5′-* GCTTCTAGACTTGTACAGCTCGTCCATGCCGCC*-3′* *,* which contained the *MluI* and *XbaI* restriction cleavage sites, respectively. The amplified fragment was first cloned into the pCR4blunt-topo vector, using TOPO cloning kit (Invitrogen), and its sequence was verified. The GFP gene in the HIV-1 Gag-iGFP vector [25] was replaced with the mCherry sequence by restriction digestion with *MluI* (New England Biolab) and *XbaI* (New England Biolab) and ligation with T4 DNA ligase (New England Biolab). To construct mCherry-ICAM-1 plasmid, the pCDM8 ICAM-1 vector (Addgene Inc., Cambridge, MA) was used as a template for the transmembrane domain of ICAM-1, and the pRSET-BmCherry (from R. Tsien, University of California, San Francisco) was used as a template for mCherry fragment. PCR products of mCherry and ICAM-1 transmembrane fragment were cloned into p3xFLAG CMV9 vector (Sigma).

### Virus Production

Pseudoviruses were produced by transfecting 10 cm dishes of HEK293T/17 cells using the PolyFect transfection reagent (Qiagen, Valencia, CA). DiD labeling was performed as described in [41]. Briefly, 18 h post-transfection, cells were incubated with Opti-MEM containing 10 µM DiD for 4 h in the CO_2_ incubator, washed and incubated for additional 24 h in a fresh growth medium. The virus-containing medium was collected at 48 h post-transfection, passed through a 0.45 µm filter, aliquoted, frozen and stored at −80°C. The infectious titer was determined by a β-Gal assay (as described in [12]) in TZM-bl cells for HIV-1 Env pseudotyped viruses and in TZM-bl/TVA950 cells for ASLV-A pseudotyped viruses.

The MLV-core-based pseudoviruses were produced by co-transfecting HEK293T/17 cells with 2 µg MLV Gag-Pol, 1 µg MLV Gag-GFP, 1 µg pcRev, 2 µg MLV LTR lacZ, and 3 µg HXB2 Env lacking the cytoplasmic tail. The HIV-1 core-based pseudoviruses were produced by co-transfecting 1 µg pR8ΔEnv, 3 µg Gag-iCherry or Gag-iGFP, 2 µg YFP-Vpr or mCherry-ICAM-1 or EcpH-ICAM-1, and 3 µg of HXB2 Env or ASLV-A Env plasmids.

### Western Blotting

Pseudoviruses co-labeled with HIV-1 Gag-iGFP and DiD were prepared as described above in the presence of a indicated concentration of SQV. Viruses were concentrated 10 times using the Lenti-X™ Concentrator (Clontech, Mountain View, CA). The amount of virus was determined by p24 ELISA, as described in [42]. Equal amounts of p24 were mixed with SDS-PAGE sample buffer (Bio-Rad, Hercules, CA) supplemented with 5% β-mercaptoethanol, boiled for 10 min at 95°C, and loaded onto a 8–16% polyacrylamide gel (Bio-Rad). Proteins were transferred onto nitrocellulose membrane, blocked, and incubated with anti-p24 183 monoclonal antibody, or Living Colors GFP monoclonal antibody (Clontech) overnight at 4°C. Rabbit anti-mouse horseradish peroxidase-conjugated antibody (Millipore, Temecula, CA) was used for protein detection by a chemiluminescence reagent from GE Healthcare. The signal was visualized on the Chemi-Doc Imager (Bio-Rad) and the densitometry analysis was done using ImageLab software (Bio-Rad).

### Real Time Imaging of Virus Fusion with Endosomes

CV-1 cells stably expressing the receptors for subgroup A ASLV (TVA950) were grown to near confluency on glass-bottom 35 mm Petri dishes (MatTek, Ashland, MA) in phenol red-free medium. Dishes were chilled on ice and centrifuged at 2,100×g (4°C) for 20 min with ∼1.5·10^4^ IU of pseudoviruses co-labeled with HIV-1 Gag-iCherry and YFP-Vpr. Unbound viruses were removed by washing, and cells were mounted onto a Personal DeltaVision microscope (Applied Precision, GE Healthcare) equipped with an environmental enclosure maintained at 37°C and an EM-CCD camera (Photometrics). Virus internalization and fusion were initiated by adding 1 ml of warm HBSS. Images were acquired using a 40×/1.3 NA (Olympus) oil immersion objective and the CFP/YFP/Cherry filter set from Chroma (Bellows Falls, VT) every 2.2 sec for 30–40 min. The axial drift caused by the change in temperature upon moving cold dishes to 37°C was compensated using the Ultimate Focus™ module of the Personal DeltaVision system.

For imaging synchronized ASLV-A fusion, CV-1 cells expressing the TVA950 receptor were grown on glass-bottom Petri dishes in phenol red-free medium. Viruses were pre-bound to cells in the cold, as described above, and cells were incubated at 37°C for 40 min in a solution containing isotonic HBSS supplemented with 2% FBS and 70 mM NH_4_Cl (pH 7.8). The cells were then transferred into a serum-free HBSS containing 70 mM NH_4_Cl and imaged at 37°C. Virus fusion with endosomes was initiated by local perfusion with HBSS using a 100 µm plastic tip and a 4 channel perfusion system (Bioscience Tools, San Diego) controlled by the SoftWorx software. After 2–3 min (as indicated), cells were perfused with 70 mM NH_4_Cl to restore neutral pH in endosomes. Imaging of synchronized fusion typically lasted 8–10 min.

### Virus Lysis with Saponin

Pseudoviruses were allowed to adhere on poly-lysine coated glass bottom Petri dishes (MatTek) or 8-chamber coverslips (Nunc Lab-Tek, Rochester, NY) for 40 min at 4°C, and unbound viruses were removed by washing. Unless otherwise indicated, virolysis was imaged at 37°C by acquiring images every 5 sec. After acquiring first three images, the acquisition was paused, an equal volume of a pre-warmed 2×saponin in HBSS was added to a final concentration of 0.1 mg/ml, and imaging was continued for ∼40 min for assessing the general kinetics of lysis or for 5 min for examining the correlation between HIV-1 maturation and the extent of lysis.

### Measurements of Endosomal and Intraviral pH

Changes in the cytosolic, endosomal and intraviral pH caused by NH_4_Cl removal were measured, as described previously [30]. In brief, pH measurements were done based on the ratio of signals from the pH-sensitive GFP (pKa 6.15 [43]) and the pH-insensitive lipophilic dye, DiD. Pseudoparticles co-labeled with GFP-ICAM-1 (the viral membrane marker) and DiD were adhered to a coverslip and exposed to buffers of different acidity. The obtained calibration curve was then used to determine the pH changes in intracellular compartments and in the virus interior. The pH within virus-carrying endosomes was measured using GFP-ICAM-1- and DiD-labeled viruses, the intraviral pH was calculated using pseudoviruses co-labeled with DiD and the viral core marker, Gag-GFP. The cytosolic pH dynamics was measured by transiently transfecting cells with GFP and co-staining with DiD.

### Image Analysis

Single virus tracking was performed with the Volocity software (Perkin Elmer, MA), as described in [12], [44]. Briefly, single fluorescent objects were identified by YFP-Vpr intensity thresholding and tracked to obtain the mean fluorescence intensities of the viral content (iCherry) and capsid (YFP-Vpr) markers over time. Pseudovirus lysis was analyzed by identifying individual particles by intensity thresholding and calculated the sum of fluorescence intensities of all particles in the image field as a function of time. In addition, individual lytic events were examined by tracking single immobilized virions using the relatively constant YFP-Vpr signal and calculating the mean fluorescence of YFP and of iCherry (content marker) signals over time. Similar analysis of iGFP-containing viruses was done using particles co-labeled with a lipophilic dye DiD, which was used to track lysis of individual particles, as described in [44]. The relative frequencies of partial, full and no lysis events were determined by analyzing the extent of iCherry or iGFP release. The ratio of mean fluorescence of a single particle at the end of a lysis experiment and the mean fluorescence prior to saponin addition was calculated. Partial release was arbitrarily defined as loss of 30–80% of the fluorescence signal (after background subtraction). Particles retaining >80% of the initial iCherry fluorescence were dubbed not lysed and those that retained <30% of their content marker were considered fully lysed.

## Results

### Release of the Viral Content Marker Correlates with Gag Processing

If, as suggested in [27], the majority or all iGFP molecules produced by proteolytic cleavage of HIV-1 Gag-iGFP is trapped within the mature “sealed” capsid, this content marker would not be fully released through a fusion pore (Fig. 1B). In this scenario, iGFP would escape from the virus after the capsid disassembly step. In contrast, mature particles with “unsealed” capsids would release iGFP in one step (Fig. 1A). To assess the pattern of iGFP escape through a pore, we lysed pseudoviruses with saponin, which forms small cholesterol-dependent pores in the viral membrane [45]. This approach previously employed by our group [30], [44] allows testing the entire virus population, instead of relying on the relatively rare, asynchronous fusion events.

**Figure 2:**
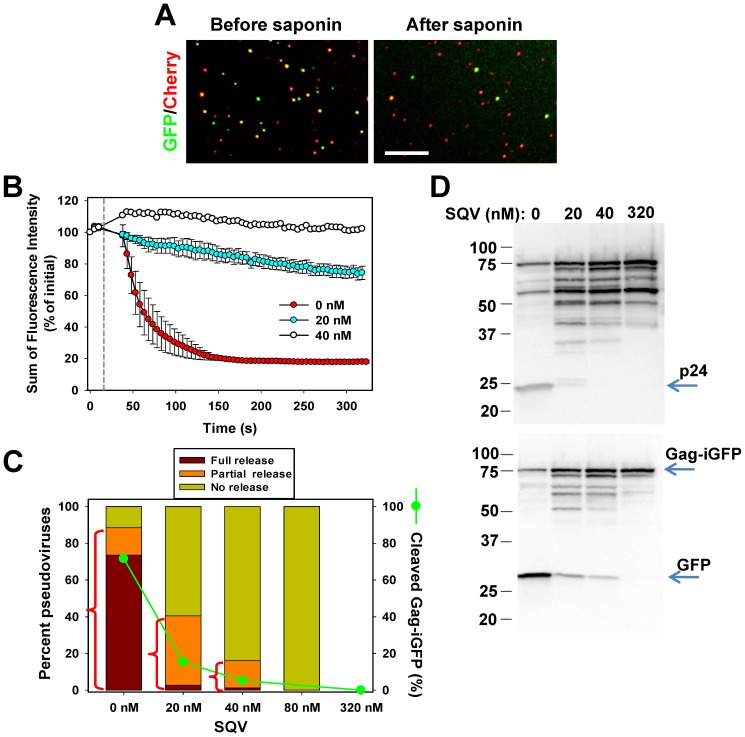
The fraction of a releasable GFP-based content marker correlates with the extent of HIV-1 maturation. (A) Lysis of pseudoviruses co-labeled with Gag-iGFP (content marker, green) and the Cherry-ICAM-1 chimera (membrane marker, red). Pseudoviruses immobilized on a coverslip (left) were exposed to saponin at 37°C (0.1 mg/ml in HBSS, right). Scale bar is 8 µm. (B) The extent and kinetics of HIV-1 pseudovirus lysis. Gag-iGFP-labeled viruses produced in the presence of escalating doses of saquinavir (SQV) were immobilized onto coverslips and imaged. After the first three images, the acquisition was paused, saponin was added at the final concentration of 0.1 mg/ml, and imaging was continued for ∼5 min at 37°C. The total fluorescence of all iGFP-positive particles as a function of time was calculated for each experiment and the average fluorescence profile from 4–5 independent experiments were plotted (error bars are SEM). The fluorescence profile for 40 nM SQV-treated viruses is the mean from 2 independent experiments. (C) Pseudoviruses co-labeled with Gag-iGFP and DiD exhibit different degrees of iGFP release upon addition of saponin, from full or partial loss of content to no iGFP release. More than 200 particles were tracked for each SQV concentration. Red brackets show the total percentage of full and partial release events. Green circles show the fraction of free iGFP relative to the total amount of fluorescent protein obtained by densitometry analysis of bands shown in panel D. (D) The effect of escalating doses of SQV on HIV-1 Gag and Gag-iGFP cleavage. Pseudoviruses produced in the presence of indicated concentrations of SQV were analyzed by SDS-PAGE and blotted with antibodies to HIV-1 Gag/p24 (top panel) or with anti-GFP antibodies (bottom panel). The four intermediate bands on the GFP blot that run between Gag-iGFP and iGFP, likely correspond to MA-GFP-CA-NC, MA-GFP-CA, GFP-CA-NC and GFP-CA.

Pseudoviruses labeled with Gag-iGFP were adhered to poly-lysine coated coverslips and imaged. Saponin was added shortly after the onset of image acquisition, and the resulting release of viral content was recorded. The number of GFP-positive puncta (Fig. 2A) and the sum of iGFP intensities for all particles in the image field (Fig. 2B) quickly decreased after addition of saponin. By contrast, pseudoviruses produced in the presence of the HIV-1 protease inhibitor, saquinavir (SQV), did not exhibit detectable content release (Fig. 2B). This demonstrates that neither inadvertent photobleaching nor virus detachment from a coverslip contributed to the loss of the iGFP signal. A similar lysis pattern was observed for pseudoviruses labeled with another “internal” fluorescent protein, Gag-iCherry, as well as for particles containing the C-terminally tagged MLV Gag-GFP, which have been used in our previous single virus fusion studies [12], [30], [44] (Fig. S1).

Analysis of single particle lysis events revealed that 74% of virions fully released their content in one quick step, 15% lost a fraction of iGFP, and the remaining 11% showed no detectable loss of content (Fig. 2C). The existence of a lysis-resistant GFP pool is consistent with the presence of unprocessed wild-type Gag and Gag-iGFP precursors in the pseudovirus preparation (Fig. 2D, first lane) and is not due to the inability of saponin to permeabilize these particles (Fig. S2). We therefore hypothesized that the extent of virolysis (release of iGFP) reflects the efficiency of Gag-iGFP processing. To investigate the relationship between HIV-1 maturation and the fraction of iGFP released by saponin, pseudoviruses were produced in the presence of escalating doses of SQV. Saponin-induced lysis of these particles was measured (Fig. 2B, C), and the efficiency of Gag-iGFP cleavage was assessed in parallel experiments by SDS-PAGE (Fig. 2D). Intermediate bands corresponding to partial Gag and Gag-iGFP cleavage products were apparent at low doses of SQV. As expected, the fraction of particles that were resistant to lysis increased with the SQV concentration, whereas the fraction of virions that fully released iGFP dropped precipitously, even at the lowest SQV concentration tested (Fig. 2C). Interestingly, a greater fraction of particles obtained in the presence of 20 nM SQV underwent partial lysis compared to control pseudoviruses. This result, along with a significant fraction of partially lysed particles produced in the presence of 40 nM SQV, argues against the iGFP entrapment model (Fig. 1B). Contrary to our data, this model predicts that the fraction of particles with fully matured capsids that retain iGFP should decrease upon partial inhibition of proteolysis.

The combined fraction of full plus partial lysis events (Fig. 2C) and the overall loss of fluorescence in the presence of saponin (Fig. 2B) were in good agreement with the extent of Gag-iGFP processing expressed as iGFP/Gag-iGFP ratio (Fig. 2C, D). This ratio was calculated by normalizing the intensity of iGFP band to the total intensity of GFP-positive bands determined by densitometry analysis (Fig. 2C, green circles). Correlation between the fractions of particles exhibiting different degrees of iGFP release and virus maturation supports the notion that iGFP release is an indicator of Gag-iGFP processing and that iGFP, but not the partial cleavage products, escapes through saponin-mediated pores. Thus, single virus lysis can be used as a surrogate assay for HIV-1 maturation. The partial HIV-1 maturation phenotype revealed by the lysis experiments is difficult to demonstrate using conventional biochemical assays. Collectively, our results imply that full or partial retention of the GFP signal is due to the lack of cleavage or incomplete cleavage of Gag-iGFP, respectively.

### The Viral Content Marker is Immediately Released through a Pore in the Viral Membrane

The quick loss of iGFP upon addition of saponin (Fig. 2B) is consistent with the notion that this marker escapes through small pores without the requirement for capsid disassembly. To verify this conclusion and to assess the relationship between pore opening to content release, we independently detected the formation of a lytic pore by imaging changes in the intraviral pH. We reasoned that the pH inside the virus must be close to neutral, owing to the lack of buffering or active ion transport. Therefore, if the surrounding pH is above 7.0, the formation of a pore in the viral membrane should raise the intraviral pH. We imaged changes in the intraviral pH caused by pore formation by incorporating YFP-Vpr into the HIV-1 core (see for example [46], [47]). The highly pH-sensitive YFP (pKa 7.1 [43]) reports the pH changes and provides a reference signal for tracking particles before and after the release of iCherry (see below). Pseudoviruses were co-labeled with Gag-iCherry in lieu of Gag-iGFP to avoid spectral overlap with YFP and, more importantly, because the Cherry fluorescence is less pH-dependent compared to GFP (pKa <4.5 vs. 6.15, respectively [43], [48]). The pH-resistance of iCherry fluorescence is essential for single virus fusion experiments described below, since it eliminates the ambiguity in interpreting the loss of signal.

**Figure 3:**
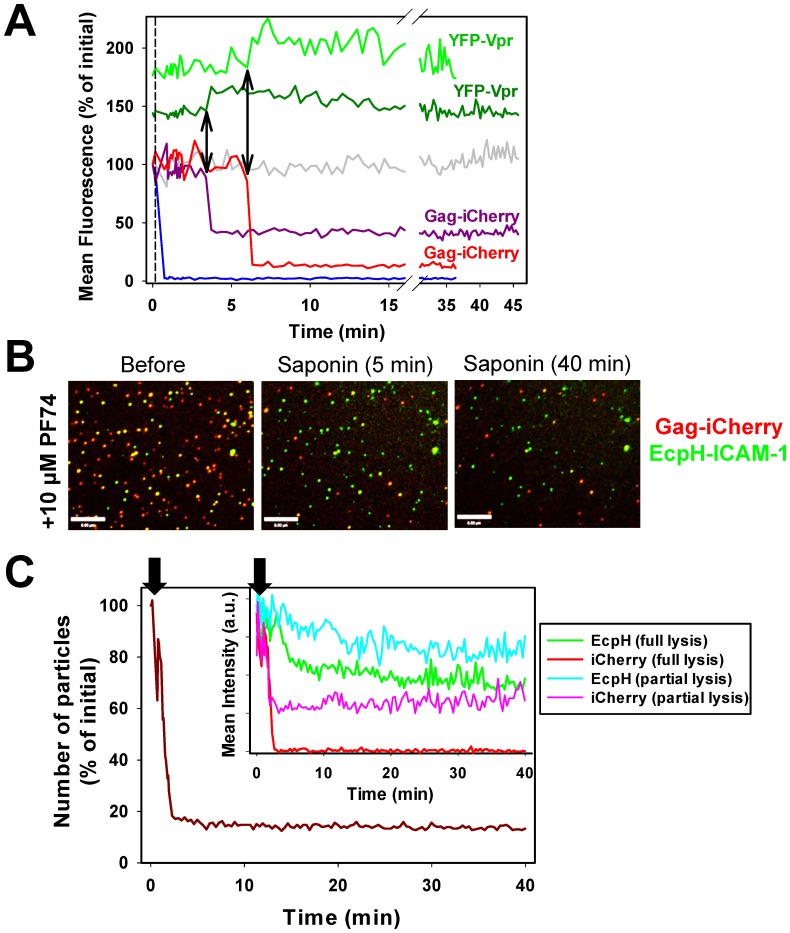
Free iCherry is released through a pore in the viral membrane, while the saponin-resistant content marker is retained under conditions expected to destabilize the capsid. (A) Content release from single pseudoviruses co-labeled with YFP-Vpr (capsid marker) and HIV-1 Gag-iCherry (content marker). Particles were adhered to a coverslip and imaged for 40 min at 37°C. Saponin was added after the third image frame (vertical dashed line). Normalized mean fluorescence intensities of iCherry and YFP from representative particles over time are plotted. Note the two distinct groupings in the graph where the YFP traces are shifted upward relative to the iCherry traces for visual clarity. The gray trace shows a particle that did not release its content marker and the blue trace corresponds to a typical particle that fully released iCherry upon addition of saponin before the imaging was resumed (the corresponding YFP traces are not shown for clarity). Red and dark pink lines show lytic events exhibiting different degrees of iCherry release; the corresponding YFP traces (green and dark green, respectively) are also shown. Double-arrows mark the onset of lysis and the corresponding increase in the YFP signal for the two representative partially lysed particles. Note that ∼10% of particles did not exhibit detectable changes in YFP fluorescence at the time of iCherry release (not shown). The lack of YFP dequenching is likely due to the slightly elevated membrane permeability, which equilibrates the pH across the viral membrane prior to addition of saponin. (B) Images of coverslip-immobilized pseudoviruses co-labeled with Gag-iCherry (red) and EcpH-ICAM-1 (green) before and after lysis with saponin. Saponin (0.1 mg/ml) was added together with 10 µM PF74 in phosphate buffer and the resulting lysis was imaged for 40 min at 37°C. Scale bar is 8 µm. (C) Single particle analysis of the lysis experiment illustrated in panel B. The number of Cherry-positive particles over time quickly decreased after adding saponin and PF74 (arrow) and stabilized after ∼3 min. *Inset*: Mean intensities of iCherry (red and pink traces) and EcpH (green and cyan traces) fluorescence as a function of time after addition of saponin (arrow) are plotted for representative full iCherry release (red) and partial release (pink) events. A slow decrease of the EcpH-ICAM-1 signal over time in panels B and C is likely related to disruption of the viral membrane upon prolonged exposure to saponin.

Analysis of single particle lysis revealed that iCherry release was completed faster than we could resolve (interval between acquisitions ∼5 s, Fig. 3A). Importantly, coincident with the loss of iCherry, most particles exhibited a significant increase in the YFP-Vpr signal. Control experiments showed that, as expected, this increase was not due to FRET between YFP and Cherry prior to lysis (data not shown). Collectively, these findings demonstrate that changes in YFP fluorescence report the formation of pores in the viral membrane through sensing the pH-difference between the viral interior (most likely neutral) and an external solution buffered at pH 7.6–7.8.

As with iGFP-labeled particles (Fig. 2C), we observed varied degree of iCherry release, from full or partial loss of signal to lack of release (Fig. 3A). Here, in order to promote capsid uncoating [49], saponin was added to viruses adhered to a coverslip, and imaging was performed for up to 40 min at 37°C. We reasoned that, if incomplete release of iCherry was caused by the capsid-entrapment, a combination of saponin and elevated temperature could facilitate uncoating and thus release the remaining content marker (Fig. 1B). However, particles undergoing partial lysis did not release the rest of the iCherry marker (Fig. 3A). Furthermore, post-permeabilization release of iCherry was not observed when saponin lysis was performed in the presence of a small-molecule inhibitor PF74 that blocks infection by destabilizing the HIV-1 capsid [32] (Fig. 3B, C). In these experiments, we used the pH-sensitive viral membrane marker, EcpH-ICAM-1 (see Materials and Methods), in order to avoid potential adverse effects of incorporation of the overexpressed YFP-Vpr on the capsid structure/stability. The fraction of saponin-resistant particles (∼15%) observed shortly after adding saponin did not decrease over time, in spite of the presence of PF74 (Fig. 3B). Single particle analysis confirmed that pseudoviruses undergoing partial lysis retained the residual iCherry signal for up to 40 min at 37°C (Fig. 3C). The ability of PF74 to inhibit HIV-1 infection in a dose-dependent manner (Fig. S3) demonstrates the functional activity of this compound *in vivo*. Considering that PF74 also destabilizes the HIV-1 capsid *in vitro* [32], the lack of effect on iCherry release from pseudoviruses argues against the capsid retention model (Fig. 1B).

Together, these results suggest that, for every particle, the extent of iCherry release is determined by the extent of cleavage of Gag-iCherry, a precursor that cannot be readily released through small pores (Fig. 1C). This model is supported by the observed correlation between the content release and Gag processing (Fig. 2B–D). Importantly, simultaneous imaging of iCherry release and of changes in the intraviral pH demonstrates that free iCherry is lost immediately after opening of a saponin pore. Our data thus imply that mature pseudoviruses have either “leaky” capsids (Fig. 1B) or sealed capsids which do not contain significant amounts of iGFP. We next applied the above imaging strategy to examine the relationship between pore formation and content release upon virus-cell fusion.

### Intraviral pH Sensing Demonstrates the Release of Viral Content Marker through a Nascent Fusion Pore

**Figure 4:**
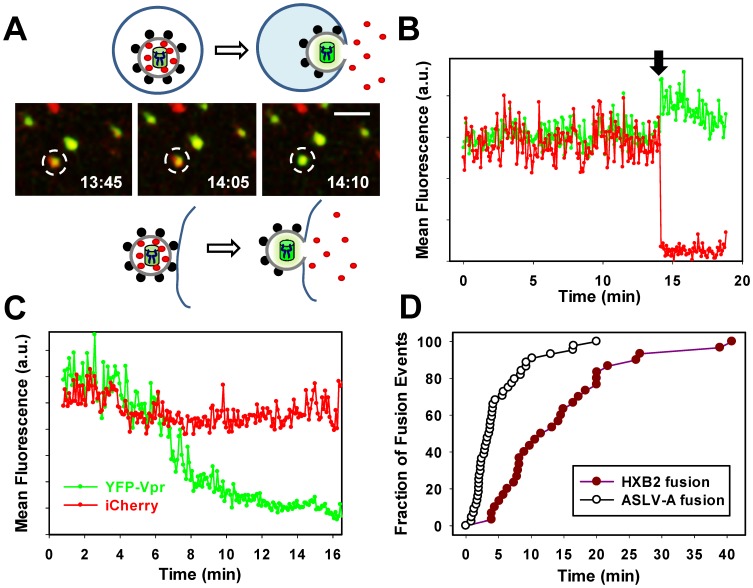
Detection of HIV-1 fusion by content release and intraviral pH-sensing assays. HIV-1 pseudoviruses bearing HXB2 Env and co-labeled with YFP-Vpr and Gag-iCherry were spinoculated onto CV-1/CD4/CXCR4 cells in the cold. Cells were washed and imaged at 37°C. (A) Images of single virus fusion leading to loss of the iCherry signal (red). Time stamps (in min:sec) indicate the time after shifting the cells to 37°C (see also movie S1). Scale bar is 2 µm. Cartoons above and below the image panel illustrate that this labeling approach does not discern between virus-endosomes (top) and virus-plasma membrane (bottom) fusion. (B) Mean fluorescence intensities of YFP and iCherry signals for the particle shown in panel A. Virus-cell fusion results in simultaneous YFP fluorescence dequenching and loss of the iCherry signal (arrow). (C) An example of a particle that fails to fuse. The slow decrease in the YFP fluorescence is caused by acidification of the virus’ interior, which most likely reflects the pH drop in endosomal compartments. (D) The kinetics of HIV-1 fusion with cells measured as the distribution of waiting times from raising the temperature to loss of iCherry (dark red circles). For comparison, the kinetic of ASLV-A Env-mediated fusion of HIV-1-based particles with CV1 cells expressing TVA950 is also shown (open circles).

To verify that loss of viral content faithfully reports fusion, HIV-1 Env pseudotyped particles co-labeled with YFP-Vpr and Gag-iCherry were pre-bound in the cold to CV-1 cells expressing CD4 and CXCR4. Virus entry and fusion were initiated by shifting to 37°C. Single particle tracking revealed that a small fraction (∼1%) of double-labeled viruses released iCherry and that these loss of content events coincided with a significant increase in the YFP signal (Fig. 4A, B and movie S1). Thus, sensing the intraviral pH permits the detection of small lytic pores (Fig. 3) and fusion pores (Fig. 4B) formed in the viral membrane. Figure 4A also illustrates that changes in the YFP-Vpr and Gag-iCherry signals do not discriminate between the virus-plasma membrane and virus-endosome fusion. Importantly, however, this labeling strategy enables simultaneous detection of fusion pores by two independent loss-of-signal (iCherry) and gain-of-signal (YFP) assays.

In contrast to fusing particles, pseudoviruses that failed to fuse exhibited a gradual decrease in the YFP signal over time without significant changes in the iCherry fluorescence (Fig. 4C). This slow decay in YFP fluorescence is likely due to virus trafficking through increasingly acidic endosomes *en route* to degradation, which results in a slow decrease of the intraviral pH. In control experiments carried out in the presence of the HIV-1 fusion inhibitor, C52L, none of the imaged 1,617 double-labeled particles released iCherry. These data demonstrate that loss of iCherry is a reliable indicator of HIV-1 Env-mediated fusion with target cells and that its fluorescence is not significantly affected by changes in the endosomal pH.

**Figure 5:**
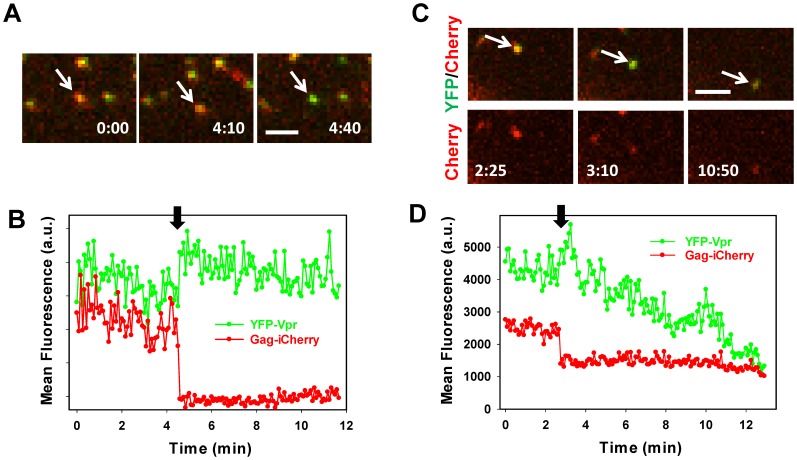
ASLV-A pseudovirus fusion with endosomes results in iCherry release and YFP dequenching. (A, C) Micrographs are showing consecutive snapshots of ASLV-A pseudovirus entry into CV1 cells expressing TVA950. Viruses were co-labeled with HIV-1 Gag-iCherry and YFP-Vpr. The release of viral content (iCherry) into the cytosol is detected by the loss of red signal at t ∼280 s (A, see also movie S2) and ∼250 s (C). The pseudovirus in panel C did not fully release its content, as better seen on the lower image panel showing only the iCherry signal. The time (min:sec) elapsed after raising the temperature is overlaid on all images. Scale bars are 2.5 µm (A) and 3 µm (C). (B, D) The intensity profiles for red (iCherry) and green (YFP) signals from the particles shown in panels A and C were obtained by single particle tracking using the YFP-Vpr channel. In both panels, iCherry release coincides with the stepwise increase in the YFP-Vpr fluorescence. The incomplete release of iCherry and the slow decrease in the YFP signal are apparent in panel D. The YFP signal decay can be caused by YFP-Vpr dissociation from the viral capsid.

To generalize the relationship between YFP-Vpr dequenching and loss of viral content, we examined another retrovirus which, unlike HIV-1, relies on low pH to undergo fusion. The subgroup A Avian Sarcoma and Leukosis Virus (ASLV-A) Env glycoprotein is activated by two sequential triggers – priming by the cognate receptor (TVA) and acidic pH [50]–[53]. Pseudotyping the HIV-1 core with ASLV-A Env increased the fusion efficiency compared to HIV-1 Env and enabled the synchronization of the fusion reaction through controlling the endosomal pH ([12], [30], [44], [54] and see below). ASLV-A pseudoviruses were pre-bound in the cold to CV-1 cells expressing the TVA950 receptor, and the temperature was raised to allow fusion. ASLV-A Env induced a quick release of iCherry from virions following entry into acidic endosomes (Fig. 5A, C and movie S2). Importantly, similar to the HIV-1 Env-mediated fusion, loss of iCherry was accompanied by dequenching of YFP fluorescence. This gain of YFP signal assay provided an independent means to detect fusion pores and confirmed that the content marker was released through a small pore. Unlike the lysis experiments, partial release of iCherry upon virus-cell fusion were not readily apparent (Fig. 5C, D), most likely because these events were more difficult to identify than complete loss of signal. Neither iCherry release nor YFP dequenching events were observed in the presence of NH_4_Cl which raises endosomal pH (data nor shown).

Analysis of individual HIV-1 Env-mediated fusion events (e.g., Fig. 4 A, B) yielded the kinetics of fusion with CV-1 cells expressing CD4 and CXCR4 (Fig. 4D). The rate of fusion with these cells was faster than with the indicator TZM-bl cells reported previously [12]. For comparison, we plotted the waiting times for the ASLV-A Env-mediated content release in CV-1/TVA950 cells (Fig. 4D). In agreement with data supporting the ASLV-A fusion with early endosomes [30], [52], the ASLV-A fusion kinetic was considerably faster than that of HIV-1 fusion. The slower rate of HIV-1 fusion may be caused by the slower internalization by CV-1 cells expressing CD4 and CXCR4 compared to the ASLV-A pseudovirus uptake through the TVA950 receptor. Taken together, studies of single virus fusion mediated by HIV-1 and ASLV-A Env reveal that iCherry escapes from virions immediately after the formation of nascent pores. The nearly instantaneous loss of iCherry shows that both viral glycoproteins tend to form relatively large fusion pores that do not restrict diffusion of the fluorescent protein.

### Loss of iCherry and YFP Dequenching are Highly Correlated

**Figure 6:**
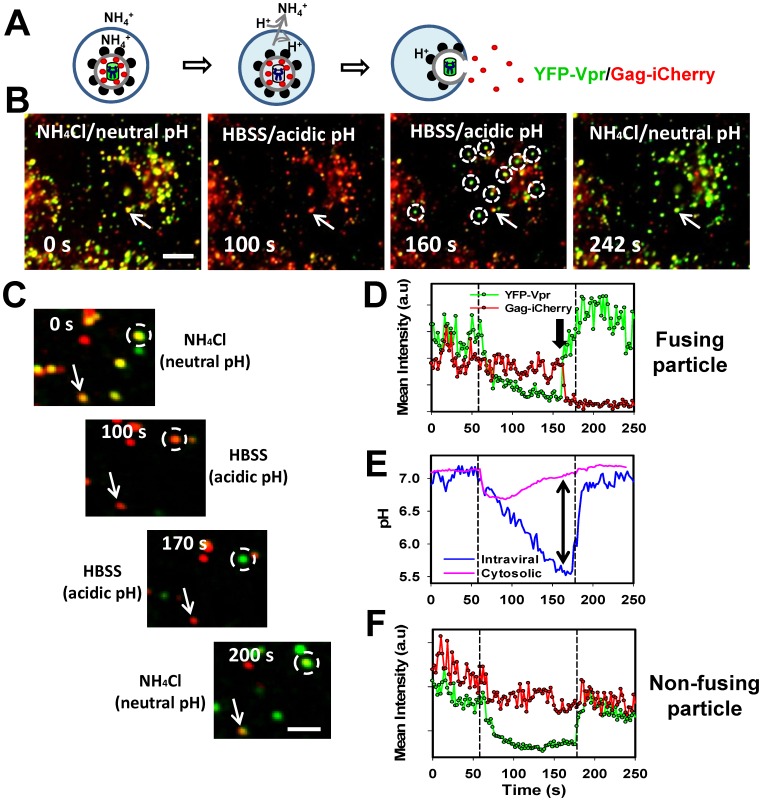
A gain-of-signal assay for detecting the synchronized fusion of NH_4_Cl-arrested ASLV-A pseudoviruses. ASLV-A pseudoviruses carrying HIV-1 Gag-iCherry and YFP-Vpr were allowed to enter CV-1 cells stably expressing TVA950 in the presence of NH_4_Cl. Virus fusion was then initiated by replacing NH_4_Cl with HBSS, thereby quickly acidifying the endosomal and intraviral pH. (A) Depiction of the NH_4_Cl arrest-release protocol for synchronized ASLV-A fusion. NH_4_Cl blocks the ASLV-A fusion and traps YFP-Vpr/Gag-iCherry labeled pseudoviruses in endosomes by raising the endosomal pH (left). Removal of NH_4_Cl results in acidification of endosomal lumen and of viral interior, as evidenced by quenching of the YFP-Vpr signal (middle). Subsequent acid-mediated fusion with an endosome results in iCherry release and YFP-Vpr dequenching caused by re-neutralization of the intraviral pH through a fusion pore that connect the virus interior to the cytoplasm (right). (B) Snapshots of ASLV-A pseudovirus fusion triggered by removal of NH_4_Cl (see also movie S3). Particles co-labeled with Gag-iCherry (red) and YFP-Vpr (green) were pre-bound to cells expressing TVA950 in the cold and incubated for at 37°C for 40 min in HBSS supplemented with 70 mM NH_4_Cl. The first micrograph is taken prior to removal of NH_4_Cl (t = 0 s). The second micrograph (t = 100 s) is taken shortly after substituting NH_4_Cl with HBSS, which results in YFP quenching due to acidification of the virus interior. The third micrograph shows pseudoviruses exhibiting the loss of iCherry and concomitant appearance of bright YFP signal caused by virus fusion (marked with white circles), while the endosomal and intraviral pH still remain acidic. The fourth micrograph shows the fluorescence pattern after returning to NH_4_Cl, which re-neutralizes the endosomal/intraviral pH and causes dequenching of the YFP signal from particles that failed to undergo fusion. The particle marked by an arrow exhibited a delayed release of iCherry relative to YFP dequenching, apparently due to slow dilation of a nascent fusion pore. Scale bar 14 µm. (C) Images of a single ASLV-A pseudovirus (dashed circle) fusing after removal of NH_4_Cl. The initial drop in the YFP signal is caused by acidification of the virus interior, whereas the loss of iCherry during the HBSS perfusion (while the intraviral pH is still acidic) corresponds to virus-endosome fusion (see movie S4). A non-fusing particle (arrow) exhibits reversible changes in YFP but not iCherry fluorescence in response to HBSS/NH_4_Cl perfusion. Scale bar is 8 µm. (D-F) The fluorescence intensity profiles for the fusing (D) and non-fusing (F) particles from panel C. The fusion event occurring during HBSS perfusion (block arrow) is manifested in the iCherry release (red) and concomitant dequenching of YFP fluorescence (green). Changes in the intraviral and the cytosolic pH during the removal/addition of NH_4_Cl determined in separate experiments are shown by blue and pink lines, respectively (E). The double-arrow in panel E shows the predicted pH difference between the intraviral and cytosolic compartments at the time of fusion shown in panels C and D.

To gain further insight into the temporal relationship between pore opening and iCherry release and to increase the gain-of-signal assay, we synchronized the ASLV-A fusion with endosomes [30]. Similar to other viruses that depend on low pH for entry, ASLV-A fusion can be arrested by NH_4_Cl which raises the endosomal pH [30], [50], [54]. Due to the extreme stability of a receptor-bound ASLV-A Env intermediate [54]–[56], the fusion arrest can be reversed by removal of NH_4_Cl, which causes quick and uniform acidification of all intracellular compartments [30] (Fig. 6A). This initial acidification is virtually independent of H^+^-ATPase [30] and is caused by excess protons released by NH_4_Cl which crosses lipid membranes in a neutral NH_3_ form [57].

Synchronized ASLV-A fusion was accomplished by first allowing the virus to enter into endosomes of target cells in the presence of NH_4_Cl for 40 min at 37°C, a condition that does not allow fusion. The dishes were mounted on a microscope stage, and NH_4_Cl was removed by local perfusion with HBSS to induce endosomal acidification and initiate the fusion reaction. We have previously demonstrated that both endosomal and intraviral pH decrease monotonically upon removal of NH_4_Cl, whereas the cytosolic pH recovers from the initial acid load within ∼2 min through an active Na^+^/H^+^ exchange ([30] and see below).

As predicted, NH_4_Cl removal induced a nearly complete loss of the YFP signal, whereas the iCherry signal remained relatively stable (Fig. 6B–F). The loss of YFP fluorescence was reversed upon replenishing NH_4_Cl. However, a large fraction of virions exhibited YFP dequenching before the end of a 2–3 min interval during which cells were perfused with HBSS (Fig. 6B and movie S3). The recovery of the YFP signal prior to returning to NH_4_Cl signifies re-neutralization of the viral interior through a fusion pore that allows equilibration of the viral and cytosolic pH (Fig. 6A).

**Figure 7:**
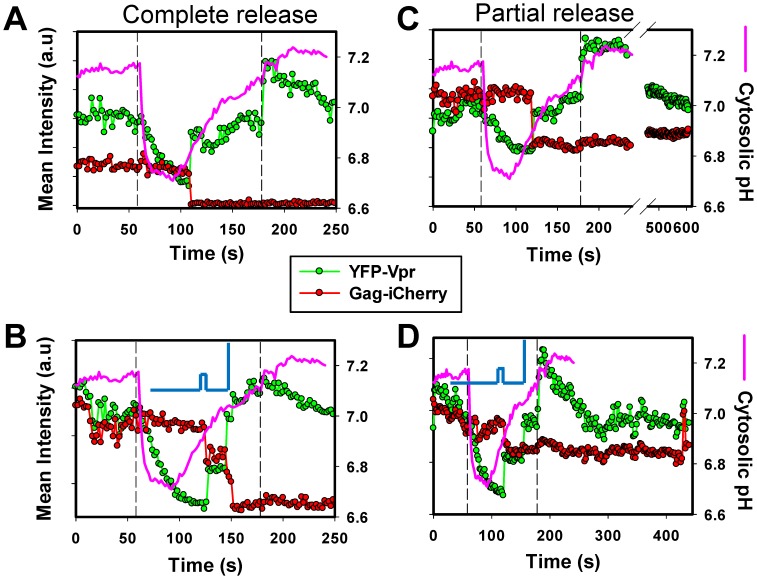
Temporal correlation between YFP dequenching and complete and partial release of iCherry upon synchronized ASLV-A pseudovirus fusion. ASLV-A fusion was arrested with NH_4_Cl and synchronously triggered by removing the weak base. (A) ASLV-A fusion results in complete release of iCherry and biphasic recovery of the YFP signal during HBSS perfusion. (B) Transient closure of a fusion pore followed by a complete loss of iCherry and recovery of the YFP signal. Pore closure was manifested in transient cessation of the drop in iCherry fluorescence and of YFP dequenching, which resumed upon pore reopening. (C) ASLV-A fusion leading to a partial release of iCherry. (D) Transient pore closure with partial release of content. The steady iCherry signal following the partial release events shown in panels B and D shows the lack of post-fusion content release. The slowly decreasing YFP signal after fusion could reflect the YFP-Vpr dissociation from the viral core. Vertical dashed lines indicate the onset and the end of HBSS perfusion. Blue lines in panels B and D illustrate the predicted profiles of flickering fusion pores.

Importantly, increases in the YFP signal during perfusion with HBSS coincided with the quick loss of iCherry (Fig. 6C, D and movies S3 and S4). Comparison of the YFP signal from fusing particles and the intraviral and cytosolic pH dynamics revealed that the pH sensor initially reported acidification of the intraviral pH (Fig. 6D, E, green circles vs. blue line). However, coincident with the iCherry release, the YFP signal exhibited a stepwise increase and started to slowly increase, mirroring the recovery of the cytosolic pH (Fig. 6E, pink line). By contrast, the YFP signal from particles that failed to fuse dropped continuously and recovered only after returning to NH_4_Cl (Fig. 6C, F). An essential advantage of synchronized ASLV-A fusion for detecting the pore formation is that the YFP-Vpr fluorescence is nearly completely quenched upon acidification of the virus interior (Figs. 6 and 7), as has been previously observed for the less pH-sensitive GFP fluorescence [30]. The loss of YFP fluorescence greatly increases the amplitude of subsequent dequenching caused by the pore formation and thus enables very sensitive detection of early fusion events (Fig. 6D and E, see also [38]). This is because the cytosolic pH does not decrease as much as the viral pH and quickly recovers from an acid load delivered upon removal of NH_4_Cl [30]. In other words, the cytoplasmic pH remains much less acidic than the viral interior. The pH differential can exceed 1 pH unit (Fig. 6E, arrow), thereby accentuating the changes in YFP fluorescence caused by fusion.

Single particle tracking further supported the remarkable temporal correlation between YFP dequenching and loss of iCherry and revealed the dynamics of pores formed upon the synchronized ASLV-A fusion (Fig. 7). A salient feature of these fusion events is the slow post-fusion increase of the YFP signal which parallels the cytosolic pH profile (see also Fig. 6D, E). Interestingly, nascent fusion pores were occasionally observed to transiently close, as evidenced by a brief cessation of YFP dequenching and iCherry decay (Fig. 7B, D). These transient pore closures typically lasted 10–20 sec and were followed by a complete recovery of YFP fluorescence and full release of iCherry. Since YFP dequenching was also arrested, transient cessation of the iCherry release must be due to the pore closure and not to a slightly delayed release of the capsid-entrapped marker. It is worth stressing that the pH-resistance of the iCherry fluorescence allows unambiguous interpretation of the pore dynamics based on its decay profile, without concerns for a significant contribution from acid-mediated quenching.

The temporal correlation between the loss of iCherry and dequenching of YFP demonstrates that iCherry escapes from virions immediately after pore opening. In agreement with the lysis experiments (Fig. 2C), about ∼15% of the synchronized fusion events resulted in partial loss of iCherry (Fig. 7C, D). The lack of post-fusion release of iCherry for as long as we were able to track the particles (up to 10 min), as well as the observed correlation between content release and Gag cleavage (Fig. 2B–D), suggest that the remaining fluorescent marker corresponds to the Gag-iCherry precursor (Fig. 1C).

In summary, the synchronized ASLV-A fusion experiments further strengthen the argument that iCherry (or iGFP) is immediately released through a nascent fusion pore and is therefore a reliable marker for viral fusion. While acidification of the viral interior during the synchronized fusion protocol can, in principle, induce premature capsid dissociation and allow complete release of the viral content through a pore, this possibility appears unlikely. Firstly, the synchronized fusion occurs within a few minutes of NH_4_Cl removal ([30] and see below). Secondly, both the loss of iCherry in the presence of saponin and the virus entry/fusion in the absence of NH_4_Cl coincide with YFP dequenching (Figs. 3A, 4 and 5), thereby ruling out the possibility that acidification of the virus interior causes premature capsid uncoating.

### YFP Dequenching can Detect Fusion in the Absence of iCherry Release

**Figure 8:**
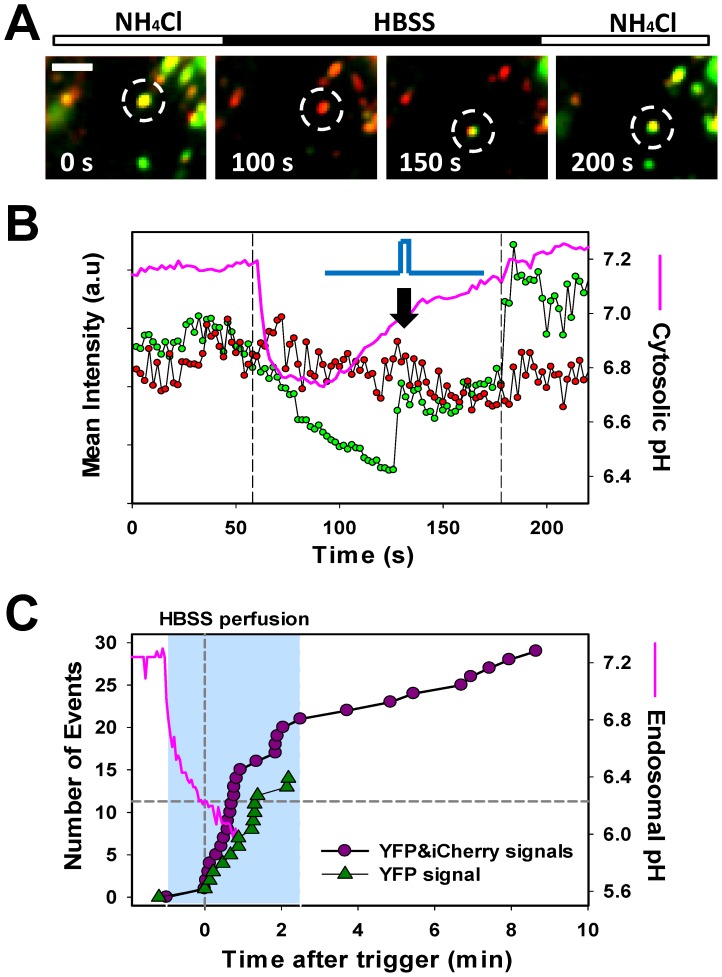
Synchronized ASLV-A Env-mediated fusion of immature HIV-1 particles can be detected by YFP dequenching. (A, B) Time-lapse images of single ASLV-A pseudovirus fusion manifested in YFP dequenching without loss of iCherry (marked by a dashed circle, see also movie S5). Scale bar is 7 µm. (B) Mean fluorescence intensity traces of YFP-Vpr (green circles) and Gag-iCherry (red circles) obtained by tacking the particle shown in panel A. Accompanying changes in the cytosolic pH are shown by a pink line. Vertical dashed lines indicate the onset and the end of HBSS perfusion. The blue trace schematically illustrates the predicted profile of a fusion pore. (C) The rates of synchronized single ASLV-A pseudovirus fusion triggered by removal of NH_4_Cl (the HBSS perfusion interval corresponds to the blue strip on the graph). The endosomal pH is shown by a pink line, and the time when the pH reaches a threshold value of 6.3 (dashed lines) and designated t = 0. Dark pink circles show the distribution of waiting times to single fusion events following the acidification of endosomal compartments below pH 6.3, as measured by simultaneous loss of the iCherry and recovery of the YFP signal. The rate of fusion events exhibiting YFP dequenching without loss of iCherry is shown by green triangles.

Whereas immature HIV-1 particles are not infectious, they can undergo fusion if pseudotyped with foreign fusion proteins (e.g., [58], [59]) or with HIV-1 Env lacking the cytoplasmic tail [58], [60], [61]. To determine whether the ASLV-A Env can mediate fusion of a small fraction of immature HIV-1 pseudoviruses present in our preparations, we monitored the synchronized YFP dequenching events that were not associated with loss of iCherry. This analysis revealed that, in agreement with the fraction of immature particles present in our preparation (Fig. 2B, D), ∼10% of particles exhibited YFP dequenching without losing iCherry (Fig. 8A, B and movie S5). This finding implies that the ASLV-A Env can induce fusion of immature HIV-1 particles.

To compare the kinetics of fusion events culminating or not culminating in iCherry release, we measured the rate of ASLV-A pseudovirus fusion induced by removal of NH_4_Cl. The earliest fusion events occurred after 30 sec from the onset of HBSS perfusion. This lag likely reflects the time required for the endosomal pH to reach 6.3, a threshold level that triggers ASLV-A fusion ([51], [52] and Fig. 8C, pink line). Therefore, to meaningfully analyze the fusion kinetics, the time point at which the endosomal pH reached 6.3 (Fig. 8C, gray dashed lines) was designated t = 0. We found that the waiting times for mature particle fusion were bi-exponentially distributed (Fig. 8C, curve fitting is not shown). The majority of fusion events were detected within ∼1 min from the point of reaching pH 6.3, while a fraction of particles fused after the endosomal pH was raised by adding NH_4_Cl. This demonstrates that the initial exposure to acidic pH triggers irreversible refolding of ASLV-A Env into a conformation that is committed to fusion at neutral pH, in agreement with our previous results [51]. Similar to early synchronized fusion events taking place upon removal of NH_4_Cl, these late events also demonstrated a striking synchrony between YFP dequenching and release of iCherry (Fig. S4). Although the kinetics of fusion events not associated with iCherry release tended to be slightly slower than fusion of mature virions, this difference was not statistically significant (P>0.602).

We then examined the dynamics of nascent fusion pores formed by immature particles by analyzing the YFP dequenching profile in the absence of iCherry release. Unlike fusion associated with simultaneous changes in the iCherry and YFP signals (Figs. 4, 5, 6, 7), the YFP signal from particles that did not lose their content only partially recovered during the HBSS pulse (Fig. 8B and Fig. S5). A further increase in fluorescence occurred only after returning to NH_4_Cl, suggesting that fusion pores formed by immature virions were short-lived and closed before the intraviral and cytosolic pH equilibrated. Together, our results imply that the kinetics of ASLV-A Env-mediated pore formation is independent of HIV-1 maturation, but that the pores formed by immature virions are unstable and tend to close. The ability to detect small and/or transient fusion pores highlights the power and sensitivity of the intraviral pH sensing assay introduced in this study.

## Discussion

Single particle tracking combined with co-labeling a virus with fluorescent content and membrane markers is a powerful means to delineate the entry pathways of enveloped viruses. We have previously employed this approach to infer the site of HIV-1 fusion based on the extent of dilution of the viral membrane marker [11], [12]. Since the overwhelming majority of the plasma membrane-bound HIV-1 pseudoviruses containing the MLV Gag-GFP released their membrane marker but not the content marker, we concluded that fusion at the cell surface did not progress beyond the hemifusion step. However, the notion that iGFP could be entrapped by the mature HIV-1 capsid [27] suggested that content release might not occur at the cell surface (Fig. 1B) and warranted further investigation. Here, through implementing a complementary technique for small pore detection, we validated the viral content release assay and showed that loss of the HIV-1 content marker faithfully reports single virus fusion. Detection of small fusion pores by sensing the intraviral pH has been previously introduced by our group [38]. In that study, we employed viruses labeled with palmitoylated YFP, which was anchored to the inner leaflet of the viral membrane. The YFP-Vpr-based pH sensor introduced in the present study offers several advantages compared to palmitoylated YFP, including more efficient incorporation and less disruptive effect on the virus assembly and fusion ([38] and data not shown).

The detection of small pores based on YFP dequenching revealed that the viral content marker was immediately released through a lytic pore or a fusion pore. Particles that retained a portion of the content marker did not release the remaining fluorescent protein under conditions expected to facilitate the capsid dissociation. These findings disfavor the model that HIV-1 pseudoviruses contain capsid-entrapped fluorescent proteins (Fig. 1B). Correlation between the extent of saponin lysis and Gag-iGFP cleavage implies that incomplete release of the viral content marker is due to partial maturation (Fig. 1C). Importantly, the capsid entrapment model predicts that the fraction of virions with mature capsid capable of entrapping iGFP should decrease in the presence of SQV. This prediction is not supported by our data (Fig. 2C). At this point, however, we cannot rule out the possibility that the capsid-confined iGFP is released after a much longer lag time than the duration of our imaging experiments. However, regardless of the reason for the lack of detectable post-fusion content release from a small fraction of virions, our results clearly demonstrate that the initial loss of iGFP marks the formation of a fusion pore.

As pointed out in the Introduction, loss of the viral lipid marker is a hallmark of fusion initiated at the cell surface: a lipophilic dye quickly redistributes to the plasma membrane and disappears [12]. Whereas HIV-1 particles were observed to exchange lipids with the plasma membrane, we have rarely detected subsequent release of the viral content. The fact that most pseudoviruses co-labeled with YFP-Vpr and Gag-iCherry contain free iCherry, which is immediately released upon opening of a small fusion pore, shows that loss of content would have been detected had the HIV-1 fused with the plasma membrane (Fig. 1). Collectively, these findings disfavor the hypothesis that full fusion at the cell surface could remain undetected owing to the capsid entrapment of iGFP (Fig. 1B). Our results thus further strengthen the conclusion that HIV-1 fails to undergo full fusion at the cell surface [12].

The pH sensing-based assay for nascent fusion pores enables studies of viral fusion in the absence of a releasable content marker (Fig. 8). We found that, whereas the waiting times to pore opening were not significantly affected by virus maturation, fusion pores formed by immature particles tended to close. Thus, although the fusion-competence of a foreign viral glycoprotein incorporated into immature HIV-1 particles may not be compromised, the immature core appears to disfavor the pore growth. Alternatively, these fusion pores could be formed by mature particles, but be too small and/or too short-lived to allow iCherry to escape. Future studies of YFP dequenching in the context of SQV-treated particles should verify the ability to detect fusion pores formed by immature particles.

The correlation between the fraction of processed Gag-iGFP and particles that completely release iGFP demonstrates that saponin lysis can be used as an express assay to test for the extent of virus maturation. Although the presence of iGFP in virions does not mean that cleavage of Gag and Gag-iGFP precursors occurred at all five conventional cleavage sites (reviewed in [62]), there is a general correspondence between the relative amounts of iGFP and p24 as a function of SQV concentration (Fig. 2D bottom and top panels, respectively). Unlike the population-based assays for Gag cleavage yielding an averaged picture of virus maturation, single particle lysis reveals the heterogeneity of viral particles. Partial release of the viral content suggests that maturation is not an all-or-none process and that not all Gag molecules within an individual particle are properly cleaved. This conclusion is supported by reports of trans-dominant inhibition of infectivity upon introducing a small fraction of cleavage-deficient Gag [63], as well as by a dramatic drop in infectivity in the presence of low doses of HIV-1 protease inhibitors that only moderately affect Gag processing [61].

In conclusion, the present study validated the use of the HIV-1 Gag-GFP based content marker for detecting virus-cell fusion. Our results demonstrate the ability of single particle imaging techniques to reliably visualize virus-cell fusion and pinpoint the sites of entry. Imaging and immunochemical data unambiguously show that the overwhelming majority of pseudoviruses labeled with Gag-iGFP or Gag-iCherry contain free fluorescent proteins that are readily released through a small pore formed in the viral membrane.

## Supporting Information

Figure S1 **Saponin lysis of pseudoviruses containing HIV-1 or MLV cores.** (A, B) Particles containing a fluorescent protein-based content marker were adhered to coverslips and exposed to 0.1 mg/ml saponin in HBSS at the time indicated by dotted lines. Virus lysis is parameterized as the number of fluorescent particles as a function of time. (A) Lysis of the HIV-1 Gag-iCherry-labeled pseudoviruses obtained in the presence of varied doses of SQV. (B) Lysis of mature and immature pseudoviruses labeled with the MLV Gag-GFP. In particles containing the MLV Gag-Pol, Gag-GFP is cleaved upon virus maturation producing a releasable nucleocapsid-GFP fragment (*Markosyan et al., Mol. Biol. Cell, 2005*). Immature pseudoviruses were obtained by transfecting producer cells with the MLV Gag-GFP plasmid without Gag-Pol.
(TIFF)

Figure S2 **Saponin permeabilizes all pseudoviruses irrespective of their maturation status.** Gag-iGFP-labeled pseudoviruses were produced in the absence or in the presence of 320 nM saquinavir (SQV), as described in Methods. Virus-containing supernatant was concentrated 10-fold, and pseudoviruses were allowed to adhere to poly-lysine-coated coverslips, washed, covered with a small volume of PBS and imaged at 37°C (A, E). Particles were then either directly exposed to pH 5.0 by adding an excess of a membrane-impermeant acidic citrate-phosphate buffer (D, H) or first permeabilized with 0.1 mg/ml saponin (B, F) and then exposed to a pH 5.0 buffer (C, G). Panels D and H show different image fields than panels A–C and E–G, respectively. Low pH-induced iGFP quenching in untreated samples is marginal (A vs. D and E vs. H), whereas addition of an acidic buffer to saponin-permeabilized viruses causes massive quenching of the iGFP signal for control (C) and SQV-treated (G) viruses, demonstrating that both mature and immature particles are permeabilized under these conditions. Scale bar is 8 µm.
(TIFF)

Figure S3 **PF74 inhibits HIV-1 infection.** TZM-bl cells grown in 96-well plates were inoculated with serial dilutions of HIV-1 HXB2 pseudoviruses in the presence of indicated concentrations of PF74 dissolved in DMEM supplemented with 10% FBS. The viruses were pre-bound to cells by centrifugation at 4°C for 30 min at 1550×*g* and their entry was initiated by shifting to 37°C. Thirty-six hours post-inoculation the extent of infection was determined by β-Gal staining, as described in Materials and Methods. The obtained viral titers were normalized to those in the absence of PF74. The results are represented as means ± STD from a single experiment performed in triplicate.
(TIFF)

Figure S4 **An example of ASLV-A Env-mediated fusion occurring after a pronounced lag following the removal of NH_4_Cl.** ASLV-A pseudoviruses co-labeled with HIV-1 Gag-iCherry and YFP-Vpr were internalized by CV-1 cells expressing TVA950 in the presence of 70 mM NH_4_Cl for 40 min at 37°C. NH_4_Cl was replaced with HBSS to trigger fusion and was replenished after 2 min. (A) Images of a particle releasing iCherry after endosomes were re-neutralized by adding NH_4_Cl. Scale bar is 8 µm. (B) Fluorescence intensity profiles for the virus shown in panel A obtained by single particle tracking. Late fusion is manifested in the loss of iCherry after the endosomal and intraviral pH were returned to neutral by perfusion with NH_4_Cl. Note the YFP signal increase at the time of iCherry release (arrow). The endosomal pH upon removal/addition of NH_4_Cl is also shown (blue line). Vertical dashed lines indicate the onset and the end of HBSS perfusion.
(TIFF)

Figure S5 **Examples of fusion without the release of iCherry.** (A, B) Fluorescence intensity profiles obtained by tracking single ASLV-A pseudoviruses labeled with HIV-1 Gag-iCherry (red) and YFP-Vpr (green). Pseudovirus fusion was arrested by incubating with CV-1/TVA950 cells in the presence of NH_4_Cl and triggered by removing the weak base. Vertical dashed lines and the thick horizontal black line mark the onset and the end of HBSS perfusion. Changes in the cytosolic pH are also shown (pink line). The points of pore opening (YFP dequenching) are marked by black arrows. The predicted pore dynamics is shown by blue lines.
(TIFF)

Movie S1 **HXB2 Env-mediated pseudovirus fusion.** Fusion of the pseudovirus (arrowhead) co-labeled with YFP-Vpr (green) and Gag-iCherry (red) with a CV-1 cell expressing CD4 and CXCR4 is manifested in loss of iCherry and slight dequenching of YFP (see Fig. 3 for more details). Scale bar is 3 µm.
(WMV)

Movie S2 **ASLV-A Env-mediated fusion with a CV-1 cell expressing the TVA950 receptor.** The Gag-iCherry signal (red) is lost while the YFP-Vpr signal (green) somewhat increases (see Fig. 4 for more details).
(WMV)

Movie S3 **Synchronized fusion of multiple ASLV-A pseudoviruses.** Fusion of pseudoviruses co-labeled with YFP-Vpr (green) and Gag-iCherry (red) with CV-1/TVA950 cells was arrested by incubation in the presence of NH_4_Cl. Perfusion with HBSS at t = 60 sec displaces the weak base, causing massive quenching of the YFP fluorescence due to acidification of the virus interior. The recovery of the YFP signal from several particles, while cells were perfused with HBSS (up until t = 180 sec) marks individual fusion events. Returning to NH_4_Cl at the end of experiment resulted in the recovery of YFP fluorescence from viruses that did not undergo fusion (see Fig. 5B for more details). Scale bar is 14 µm.
(WMV)

Movie S4 **Single synchronized ASLV-A fusion with a CV-1/TVA950 cell.** Single pseudovirus fusion (see also Fig. 5C) leads to the loss of iCherry signal (red) and increase in the YFP signal (green). The “trailing” effect manifested in transient separation of the co-localized green and red channels during quick displacement of a particle is caused by sequential acquisition of the two channels. Scale bar is 8 µm.
(WMV)

Movie S5 **Synchronized ASLV-A pseudovirus fusion detected by YFP dequenching without the release of iCherry.** A pseudovirus labeled with YFP-Vpr (green) and Gag-iCherry (red) shows dequenching of the YFP signal shortly after removal of NH_4_Cl. The iCherry signal does not change significantly during the course of the experiment. Scale bar is 4 µm.
(WMV)
