# Supplementary figures and images for: Fusion of Mature HIV-1 Particles Leads to Complete Release of a Gag-GFP-Based Content Marker and Raises the Intraviral pH
Source: PLoS One. 2013 Aug 9;8(8):e71002. doi: 10.1371/journal.pone.0071002 (PMC3739801; doi:10.1371/journal.pone.0071002)

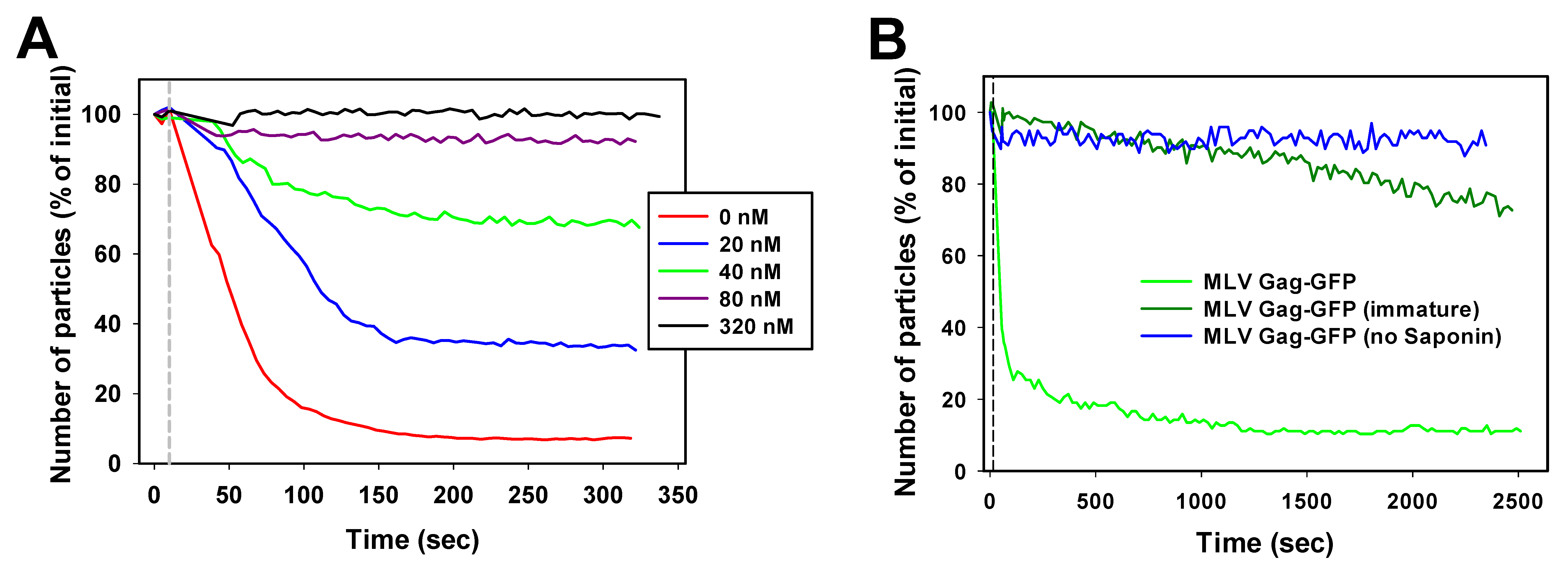

Supplement: Figure S1 — Saponin lysis of pseudoviruses containing HIV-1 or MLV cores. (A, B) Particles containing a fluorescent protein-based content marker were adhered to coverslips and exposed to 0.1 mg/ml saponin in HBSS at the time indicated by dotted lines. Virus lysis is parameterized as the number of fluorescent particles as a function of time. (A) Lysis of the HIV-1 Gag-iCherry-labeled pseudoviruses obtained in the presence of varied doses of SQV. (B) Lysis of mature and immature pseudoviruses labeled with the MLV Gag-GFP. In particles containing the MLV Gag-Pol, Gag-GFP is cleaved upon virus maturation producing a releasable nucleocapsid-GFP fragment (Markosyan et al., Mol. Biol. Cell, 2005). Immature pseudoviruses were obtained by transfecting producer cells with the MLV Gag-GFP plasmid without Gag-Pol. (TIFF) [file pone.0071002.s001.tiff]

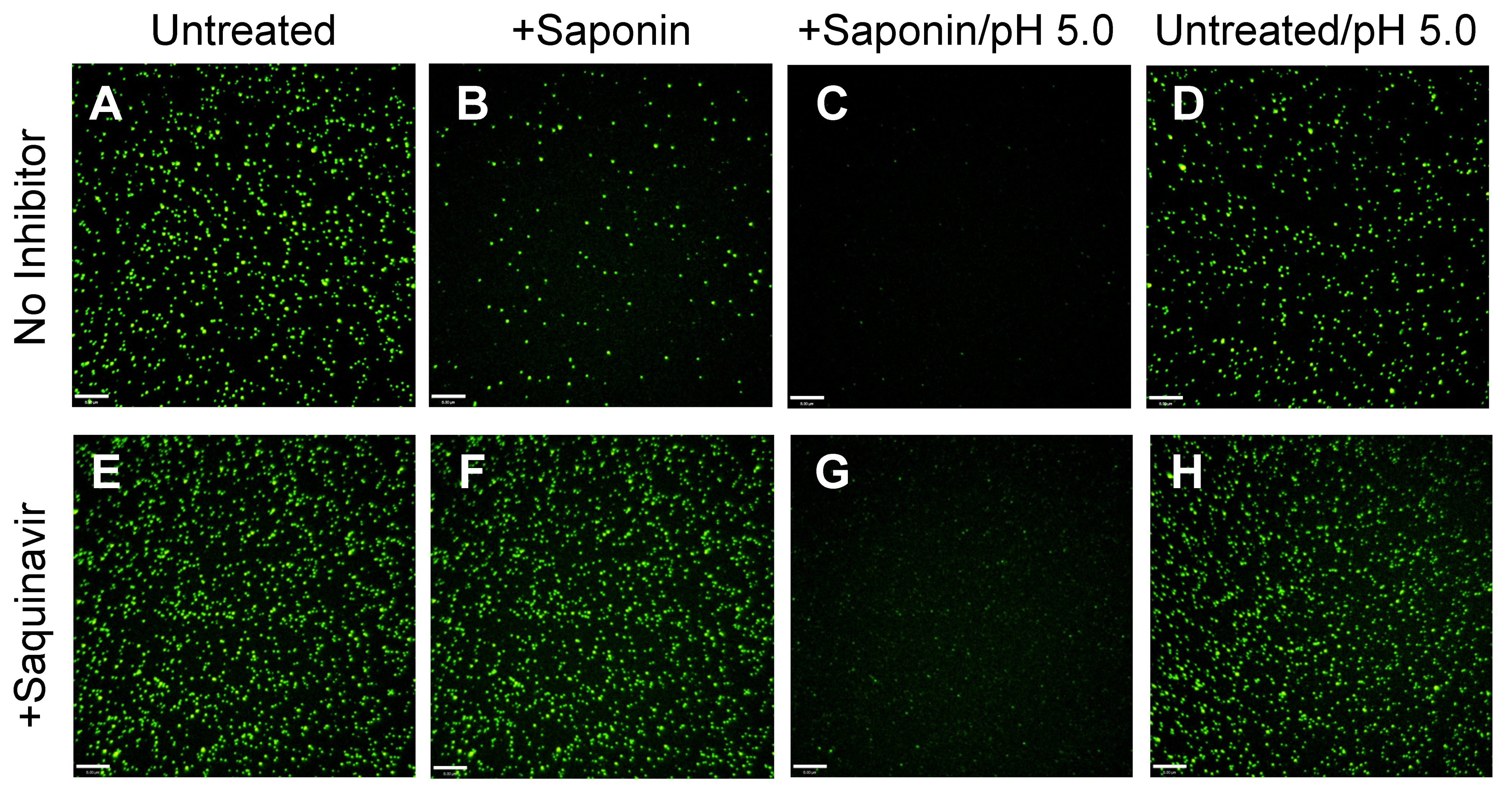

Supplement: Figure S2 — Saponin permeabilizes all pseudoviruses irrespective of their maturation status. Gag-iGFP-labeled pseudoviruses were produced in the absence or in the presence of 320 nM saquinavir (SQV), as described in Methods. Virus-containing supernatant was concentrated 10-fold, and pseudoviruses were allowed to adhere to poly-lysine-coated coverslips, washed, covered with a small volume of PBS and imaged at 37°C (A, E). Particles were then either directly exposed to pH 5.0 by adding an excess of a membrane-impermeant acidic citrate-phosphate buffer (D, H) or first permeabilized with 0.1 mg/ml saponin (B, F) and then exposed to a pH 5.0 buffer (C, G). Panels D and H show different image fields than panels A–C and E–G, respectively. Low pH-induced iGFP quenching in untreated samples is marginal (A vs. D and E vs. H), whereas addition of an acidic buffer to saponin-permeabilized viruses causes massive quenching of the iGFP signal for control (C) and SQV-treated (G) viruses, demonstrating that both mature and immature particles are permeabilized under these conditions. Scale bar is 8 µm. (TIFF) [file pone.0071002.s002.tiff]

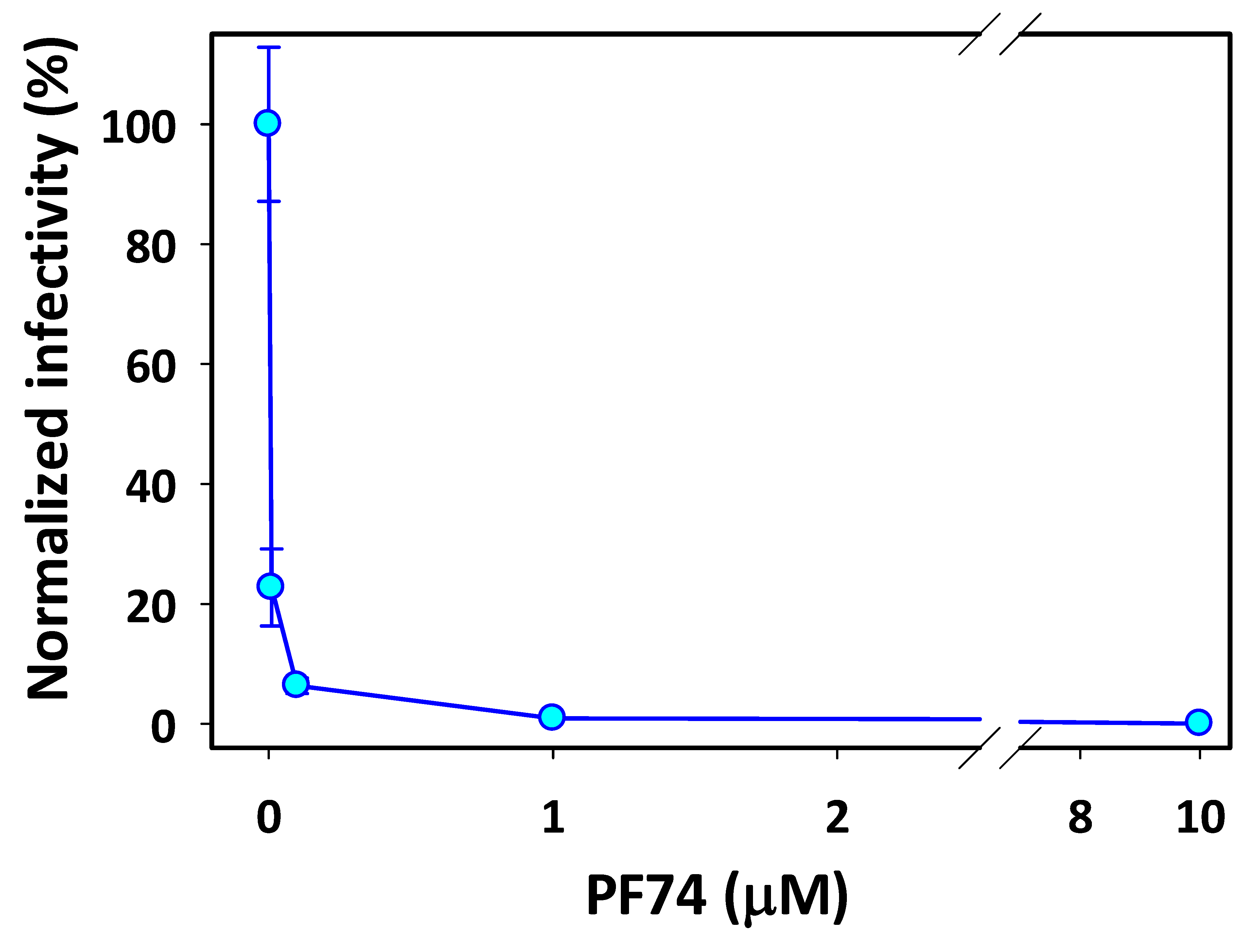

Supplement: Figure S3 — PF74 inhibits HIV-1 infection. TZM-bl cells grown in 96-well plates were inoculated with serial dilutions of HIV-1 HXB2 pseudoviruses in the presence of indicated concentrations of PF74 dissolved in DMEM supplemented with 10% FBS. The viruses were pre-bound to cells by centrifugation at 4°C for 30 min at 1550×g and their entry was initiated by shifting to 37°C. Thirty-six hours post-inoculation the extent of infection was determined by β-Gal staining, as described in Materials and Methods. The obtained viral titers were normalized to those in the absence of PF74. The results are represented as means ± STD from a single experiment performed in triplicate. (TIFF) [file pone.0071002.s003.tiff]

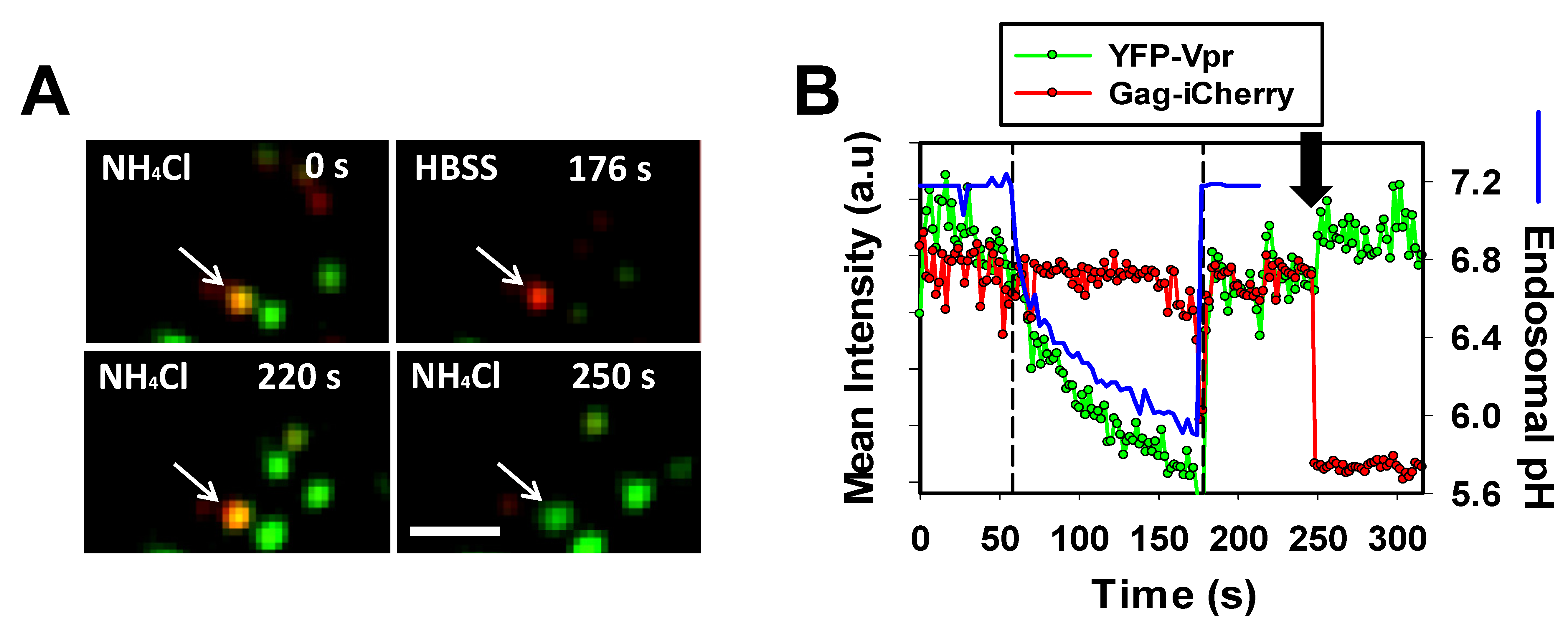

Supplement: Figure S4 — An example of ASLV-A Env-mediated fusion occurring after a pronounced lag following the removal of NH4Cl. ASLV-A pseudoviruses co-labeled with HIV-1 Gag-iCherry and YFP-Vpr were internalized by CV-1 cells expressing TVA950 in the presence of 70 mM NH4Cl for 40 min at 37°C. NH4Cl was replaced with HBSS to trigger fusion and was replenished after 2 min. (A) Images of a particle releasing iCherry after endosomes were re-neutralized by adding NH4Cl. Scale bar is 8 µm. (B) Fluorescence intensity profiles for the virus shown in panel A obtained by single particle tracking. Late fusion is manifested in the loss of iCherry after the endosomal and intraviral pH were returned to neutral by perfusion with NH4Cl. Note the YFP signal increase at the time of iCherry release (arrow). The endosomal pH upon removal/addition of NH4Cl is also shown (blue line). Vertical dashed lines indicate the onset and the end of HBSS perfusion. (TIFF) [file pone.0071002.s004.tiff]

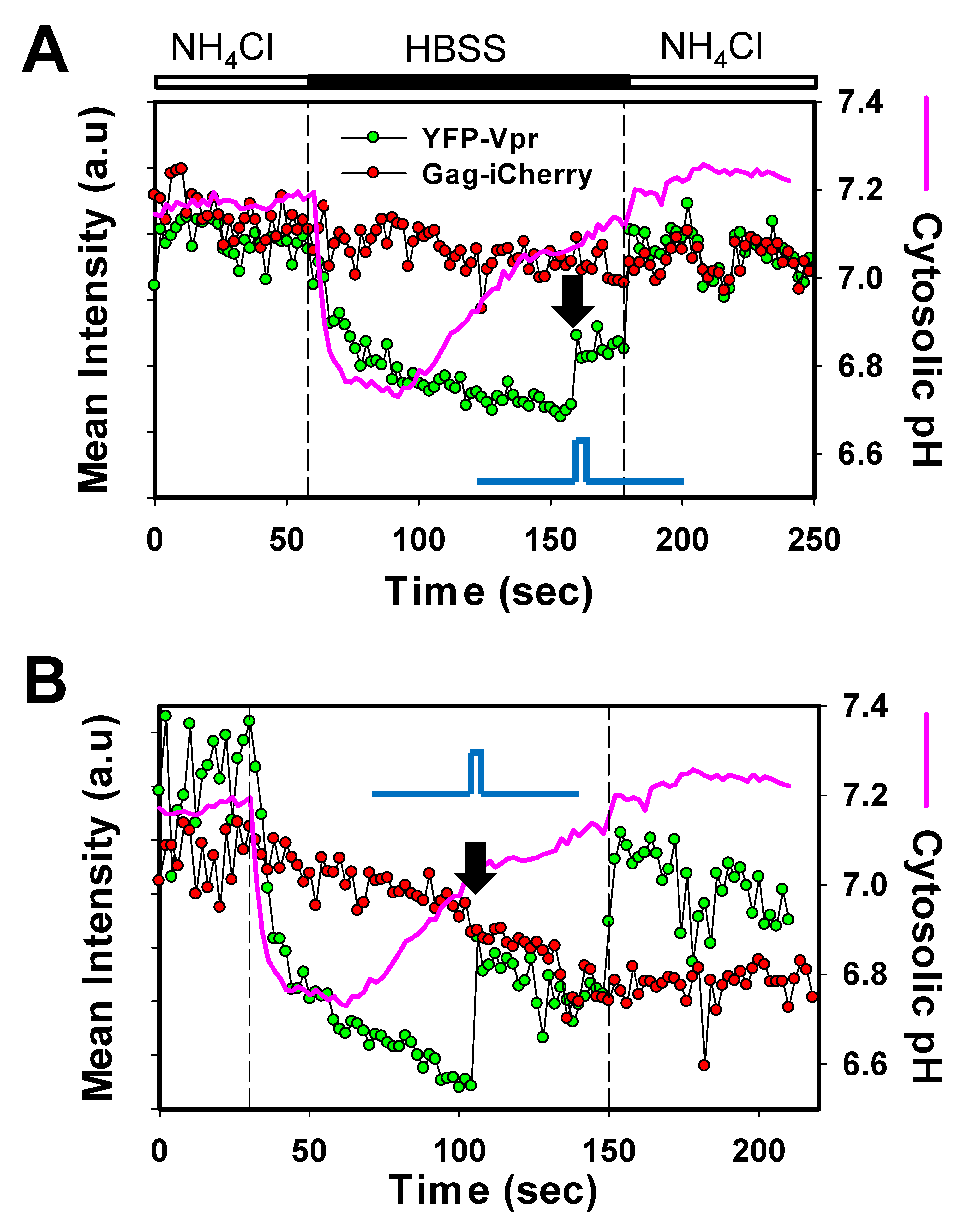

Supplement: Figure S5 — Examples of fusion without the release of iCherry. (A, B) Fluorescence intensity profiles obtained by tracking single ASLV-A pseudoviruses labeled with HIV-1 Gag-iCherry (red) and YFP-Vpr (green). Pseudovirus fusion was arrested by incubating with CV-1/TVA950 cells in the presence of NH4Cl and triggered by removing the weak base. Vertical dashed lines and the thick horizontal black line mark the onset and the end of HBSS perfusion. Changes in the cytosolic pH are also shown (pink line). The points of pore opening (YFP dequenching) are marked by black arrows. The predicted pore dynamics is shown by blue lines. (TIFF) [file pone.0071002.s005.tiff]
